# ZNF326 promotes malignant phenotype of glioma by up-regulating HDAC7 expression and activating Wnt pathway

**DOI:** 10.1186/s13046-019-1031-4

**Published:** 2019-01-28

**Authors:** Xinmiao Yu, Minghao Wang, Jingjing Wu, Qiang Han, Xiupeng Zhang

**Affiliations:** 1 Department of Surgical Oncology and Breast Surgery, First Affiliated Hospital of China Medical University, Shenyang, China; 2 Department of Neurosurgery, First Affiliated Hospital of China Medical University, Shenyang, 110001 China; 3 Department of Pathology, First Affiliated Hospital of Fujian Medical University, Fuzhou, China; 4 Department of Pathology, College of Basic Medical Sciences, and First Affiliated Hospital of China Medical University, Shenyang, China

**Keywords:** ZNF326, Glioma, Wnt pathway, HDAC7, β-catenin

## Abstract

**Background:**

Zinc-finger protein-326 (ZNF326) was initially found in the NIH3T3 cell line to regulate cell growth, however, the expression and underlying role of ZNF326 in human tumours, especially in glioma, is not fully understood.

**Methods:**

Immunohistochemistry was applied to detect the expression of ZNF326 in glioma tissues, and statistical analysis was used to analyse the relationship between ZNF326 expression and clinicopathological factors. The effect of ZNF326 on glioma cells proliferation and invasion was conducted by functional experiments both in vivo and in vitro. Chromatin immunoprecipitation and dual-luciferase assays were performed to demonstrate that histone deacetylase enzyme-7 (HDAC7) is the target gene of ZNF326. Immunoblotting, real-time PCR, GST-pulldown and co-immunoprecipitation assays were used to clarify the underlying role of ZNF326 on Wnt pathway activation.

**Results:**

High nuclear expression of ZNF326 was observed in glioma cell lines and tissues, and closely related with advanced tumour grade in the patients. Moreover, ectopic ZNF326 expression promoted the proliferation and invasiveness of glioma cells. Mechanistically, ZNF326 could activate *HDAC7* transcription by binding to a specific promoter region via its transcriptional activation domain and zinc-finger structures. The interaction of the up-regulated HDAC7 with β-catenin led to a decrease in β-catenin acetylation level at Lys-49, followed by a decrease in β-catenin phosphorylation level at Ser-45. These changes in β-catenin posttranscriptional modification levels promoted its redistribution and import into the nucleus. Additionally, ZNF326 directly associated with β-catenin in the nucleus, and enhanced the binding of β-catenin to TCF-4, serving as a co-activator in stimulating Wnt pathway.

**Conclusions:**

Our findings elucidated ZNF326 promotes the malignant phenotype of human glioma via ZNF326-HDAC7-β-catenin signalling. This study reveals the vital role and mechanism of ZNF326 in the malignant progression of glioma, and provides the reference for finding biomarkers and therapeutic targets for glioma.

**Electronic supplementary material:**

The online version of this article (10.1186/s13046-019-1031-4) contains supplementary material, which is available to authorized users.

## Background

Glioma is the most common intracranial tumour originating from the epithelial cells of the central nervous system, accounting for more than 60% of primary brain tumours [1–3]. Current therapeutic strategies for glioma consist of neurosurgical resection, chemotherapy, and radiotherapy. However, all these strategies have failed to yield an expected good prognosis of malignant glioma. This could be due to the highly aggressive nature of glioma cells that are capable of infiltrating into the adjacent normal brain tissue [4, 5]. Therefore, there is always an urgency to develop novel strategies for timely diagnosis and therapeutic agents for patients with glioma.

The Wnt pathway elicits an important regulatory signal that is able to influence embryonic development of different tissues and organs, including the nervous system. Dysregulation of the Wnt signal is implicated in the development and progression of glioma [6, 7]. Indeed, several studies have revealed that the expression and nuclear localisation of β-catenin and its transcription factor TCF4 are significantly higher in glioma compared to that in normal brain tissue, and these characteristics positively correlate with the glioma grade [8]. Similarly elevated expression of two Wnt pathway activators, TCF4 and SOX, were also reported [9, 10]. Moreover, high levels of canonical Wnt factors such as DKK1, FZD1, and LEF1 were found to be associated with very poor clinical outcome [11]. Oncogenic activities such as proliferation, inhibition of apoptosis, and invasion have also been coupled to abnormal Wnt/β-catenin signalling in glioma cell lines [12, 13]. Altogether, these findings indicate that the Wnt pathway plays a fundamental role in gliomagenesis.

In the canonical Wnt pathway, β-catenin, which is a central player of this signalling cascade, is entrapped into a protein complex formed by Axin1, glycogen synthase kinase-3β (GSK-3β), casein kinase 1α (CK1α), and adenomatous polyposis coli (APC). This complex favours β-catenin degradation by proteasomal ubiquitination. However, when a Wnt protein binds to the membrane receptors of the frizzled (FZD) and low-density lipoprotein receptor-related protein (LRP5/6) families, the degradation complex appears to be inhibited. Thus, unphosphorylated β-catenin accumulates, enters the nucleus and binds to TCF-4, and activates expression of Wnt signalling pathway target genes such as *AXIN2*, *C-MYC*, *CCND1*, and *MMP7* [14–18].

Zinc-finger protein-326 (ZNF326) was first identified in NIH3T3 cells and is believed to play an important role in neuronal differentiation [19]. Although the molecular mechanism of ZNF326 is not yet completely understood, it is essentially a protein molecule of 582 amino acids, with C2H2 zinc-finger domain, and acts as a potential transcription factor. The main functional domains include: a transcriptional activation domain (TAD) near the N-terminus (1-124aa), an intra-nuclear localisation sequence between 242-260aa (NLS), and a central region containing two C2H2 zinc-finger domains (313-336aa and 407-430aa) [20]. Until date, the expression of ZNF326 in human glioma, its effect on the malignant phenotype of glioma cells, and the possible signal transduction pathway involved have not been reported.

In this study, we report the clinical relevance of ZNF326 in glioma and its regulatory effect on the Wnt/β-catenin signalling pathway. Initially we measured the expression level of ZNF326 in human resected glioma specimens, and analysed the relationship between ZNF326 expression and clinicopathological factors of glioma. We also investigated the effects of ZNF326 on the proliferation and invasiveness of glioma cells. At the molecular level, a series of ZNF326 mutants were constructed and studied. ChIP-seq, luciferase reporter assay, and co-immunoprecipitation were applied to detect the effects of ZNF326 on the transcriptional activity of target genes. The results here provide a theoretical and experimental basis for glioma treatment strategies.

## Methods

### Human glioma tissue collection and ethics statements

A total of 133 tumour specimens (GradeI-IV) were obtained from patients (average age: 50 years) during surgery at the First Affiliated Hospital of China Medical University from 2008 to 2017. All patients were chemotherapy- and radiotherapy-nave prior to this surgical resection. The clinical data such as age, gender, tumour location and WHO grade were recorded for statistical analysis. All clinical investigations were conducted according to the principles expressed in the Declaration of Helsinki. Written informed consent was obtained from all patients, and all procedures were approved by the Institute Research Ethics Committee of China Medical University.

### Histology analysis

Assays were performed as described previously [21]. Briefly, the tissue sections were incubated with anti-ZNF326 and HDAC7 mouse monoclonal antibody (sc-390,606 and sc-74,563, respectively 1:50 and 1:20, Santa Cruz Biotechnology Inc. CA, USA). The intensity of ZNF326 and HDAC7 staining was scored as follows: 0 (no staining and weak), 1 (moderate), or 2 (strong). Percentage scores were assigned as follows: 1 (1–25%), 2 (26–50%), 3 (51–75%), and 4 (76–100%). The scores of each tumour sample were multiplied to give a final score of 0 to 8. According to the staining intensity and the staining extent scores, the immunohistochemistry (IHC) result was classified as follows: 0–3, negative (−); 4–5, weakly positive (+); 6–7, moderately positive (++) and ≥ 7, strongly positive (+++). PBS and goat serum were used as negative controls.

### Cell lines and cell culture

Human glioma cell lines, including U251, U87, SHG44 and U118 were purchased from the American Type Culture Collection (Manassas, VA, USA). All culture media were supplemented with 10% foetal bovine serum (FBS, Hyclone, Logan, UT, USA). Normal human astrocyte (NHA) is a gift supplied by Professor Anhua Wu (China Medical University). U87 and NHA was cultured in Eagle’s Minimum Essential Medium (MEM, Hyclone), while U251, U118, and SHG44 cells were all cultured in Dulbecco’s modified Eagle’s medium (DMEM, Hyclone). Cells were maintained at 37 °C in a humidified atmosphere of 5% CO_2_. All cell lines were authenticated by short tandem repeat (STR) DNA profiling.

### Plasmids and reagents

pCMV6 empty vector (#PS-100001) and Myc/DDK-pCMV6-ZNF326 (#RC-210339) were purchased from Origene (Rockville, MD, USA). pcDNA3.1 empty vector (#52535), pcDNA3.1-FLAG-HDAC7 (#13824), TCF4 plasmid (#16512), pEGFP-N1 empty vector (#86776), pEGFP-N1-β-catenin (#71367) and Super8 × TOPflash (#12456) were purchased from Addgene (Cambridge, MA, USA). pRL-TK (#E2241) was purchased from Promega (Madison, Wisconsin, USA). Control siRNA (sc-37,007), siRNA-ZNF326 (sc-88,338) and siRNA-HDAC7 (sc-35,546) were purchased from Santa Cruz Technology. The nucleotide sequence shRNA-ZNF326 was provided by Dr. Roberto Rangel and Professor Nancy A. Jenkins at the Anderson Cancer Center of the US. ShRNA-ZNF326, shRNA-HDAC7 plasmid and the lentivirus envelope shZNF326/ZNF326 were constructed by Genechem company (Shanghai, China). The mutants ZNF326-△TAD, ZNF326-△Zn1, ZNF326-△Zn2 and ZNF326-△TAD&△Zn1 + 2 were also constructed by Genechem. HA-CBP plasmid is a gift from Prof. Liu Cao (Department of Translational Medicine, China Medical University). Lipofectamine 3000 (Invitrogen, Carlsbad, CA, USA) transfection reagent was used for plasmid transfection. Puromycin (Sigma-Aldrich, St. Louis, MO, USA) was used to select stably transfected cells.

### Cell extraction and immunoblotting

Assays were performed as described previously [21]. Trichostatin A (TSA) was purchased from Sigma-Aldrich. Expression was quantified using densitometry and ImageJ software. Detailed information about primary antibody is provided in the Additional file 1.

### Immunofluorescence staining

Cells were fixed with 4% paraformaldehyde, blocked with 3% BSA, and incubated with anti-β-catenin antibody (1:100, BD Biosciences, #610153) overnight at 4 °C, followed by incubation with fluorescein isothiocyanate (FITC)-conjugated secondary antibodies, at room temperature for 1 h. Cells were counterstained with 4′,6-diamidino-2-phenylindole (DAPI). Confocal microscopy was performed using a Radiance 2000 laser scanning confocal microscope (Carl Zeiss, Thornwood, NY, USA).

### GST-pulldown assay

GST (glutathione S transferase)-conjugated β-catenin protein was expressed in *E. coli* BL21 cells and was purified using standard protocols. Glutathione-Sepharose beads (GE Healthcare, Waukesha, WI, USA) coupled with either GST or with the GST-β-catenin purified protein were incubated with the HEK293 cells lysates transfected with Myc/DDK-pCMV6-ZNF326 plasmid overnight at 4 °C. Complexes were washed and subjected to immunoblotting and coomassie blue staining.

### Chromatin immunoprecipitation and sequencing

ZNF326-overexpressing H1299 cells were crosslinked and lysed by ultrasound treatment. The pyrolysis liquid was divided into four groups and treated with different antibodies. Protein G agarose was added at 4 °C, followed by rotation for 1 h. The corresponding antibody was then added, followed by incubation overnight at 4 °C. After protein/DNA complex elution, the complex was disintegrated. After recovery of DNA samples, high-throughput sequencing was performed (#17–371; Millipore, Burlington, MA, USA).

### ChIP-qPCR assays

ChIP experiment was carried out according to the procedure described by manufacturer (Millipore, MA, USA). The immunoprecipitated DNAs were amplified by qPCR. The primers used are listed in Additional file 1.

### Dual-luciferase assay

Assays were performed as described previously [21]. Briefly, recombinant human Wnt3a (#5036-WN, R&D Systems, France) was reconstituted in PBS containing 0.2% BSA to a concentration of 10 μg/mL and used in experiments at a final concentration of 100 ng/mL. Luciferase-reporter plasmids are described in detail in Additional file 1.

### RNA extraction and real-time RT-PCR

RT-PCR assays were performed as described previously [21]. The relative transcript levels of genes were normalised to GAPDH mRNA levels, and the primer sequences are listed in Additional file 1.

### Colony formation, Matrigel invasion, and MTT assays

Glioma cells were seeded in 6-cm cell culture dish (1000 per dish) and incubated for 14 days. The plates were then washed with PBS and stained with Giemsa before counting the number of colonies consisting of > 50 cells.

Matrigel invasion and MTT assays were used in this study. All experiments were performed in triplicate. The detailed protocol is provided in Additional file 1.

### Transplantation of tumour cells into nude mice

The nude mice used in this study were treated by following the experimental animal ethics guidelines issued at China Medical University. The study was approved by Institutional Animal Research Committee of China Medical University. The nude mice (BALB/c, SPF grade, 16–18 g, 4 weeks old, and female) were purchased from Charles River (Beijing, China), and the axilla of each mouse was subcutaneously inoculated with 5 × 10^6^ glioma cells in 0.2 mL of sterile PBS. Six weeks after inoculation, the mice were euthanised and autopsied to examine tumour growth. A portion of explant tumour was fixed in 4% formaldehyde and embedded in paraffin. Serial 4-μm-thick sections were cut and stained by IHC, and the stained sections were examined under a microscope. The proliferation rate was evaluated by counting Ki-67-positive nuclei in more than 30 high-power fields (HPFs) per group.

### Statistical analysis

The statistical software SPSS 22.0 (SPSS, Chicago, IL, USA) was used for all analyses. The chi-squared test was used to assess correlations between ZNF326 expression and clinicopathological factors. Differences between the groups were tested by Student’s *t*-test. The correlation between ZNF326 and HDAC7 expression in glioma specimens was tested by *Pearson*-correlation test. A *p* value of < 0.05 was considered to indicate statistically significant differences.

## Results

### ZNF326 is highly expressed in glioma and positively correlated with tumour grade

**Fig. 1:**
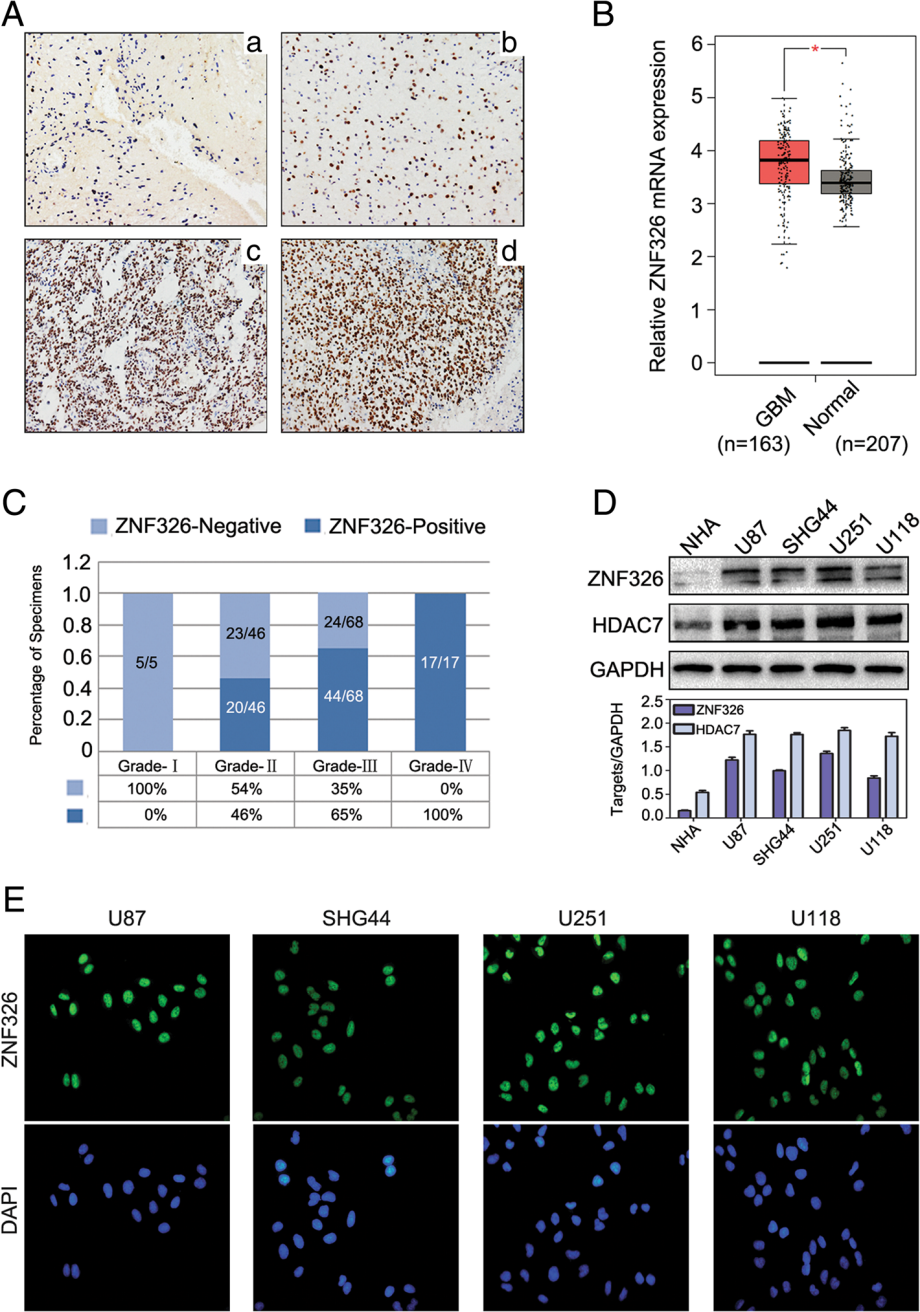
Expression and localisation of ZNF326 in glioma tissues and cell lines. **a** ZNF326 was negative in pilocytic astrocytoma, ZNF326-positive nuclear staining percent/HPF: < 1%, GradeI, (A-a, 400×), and positive nuclear expression (+) in diffuse astrocytoma, ZNF326-positive nuclear staining percent/HPF: 25%, grade II, (A-b, 400×); strongly positive expression (++ to +++) in anaplasia astrocytoma, ZNF326-positive nuclear staining percent/HPF: 75%, grade III (A-c, 400×) and glioblastoma, ZNF326-positive nuclear staining percent/HPF: > 90%, grade IV,(A-d, 400×). **b** ZNF326 mRNA expression in glioma and normal brain tissues analysed by TCGA database (*P* < 0.05). **c** The statistical view of positive expression of ZNF326 in gliomas and the positive staining percentage in different grades. **d** ZNF326 and HDAC7 expression was detected in a panel of four glioma cell lines and normal human astrocyte (NHA), using immunoblotting, GAPDH served as a loading control. **e** Immunofluorescence showed the expression and subcellular localization of ZNF326

To explore a potential role of ZNF326 in glioma tumorigenesis, we performed IHC in a cohort of 133 human patients with glioma samples to examine the expression profiles of ZNF326. We found that nearly 60.9% (81/133) of patients with glioma had high level of nuclear ZNF326 (+, ++ and +++) in the glioma samples. In addition, ZNF326 was negative in 5 cases of glioma tissues with grade-I and the ZNF326 staining was significantly associated with tumour grade (Fig. 1a, and c, *P* = 0.000) and age (*P* = 0.012), but not with gender and tumour location (Table 1). Consistently, the TCGA database (http://gepia.cancer-pku.cn/) indicated that ZNF326 mRNA levels in glioma are significantly higher than those in normal brain (Fig. 1b, *P* < 0.05). Similar results were obtained in a panel of four glioma cell lines in vitro, compared with that in normal human astrocyte cell line NHA (Fig. 1d). Immunofluorescence assay indicated ZND326 located in the nucleus of glioma cells (Fig. 1e). Altogether, these results suggest that ZNF326 could be used as a potential predictor of malignancy in gliomas.

**Table 1:** Association between ZNF326 expression and clinicopathologic characteristics in 133 glioma specimens

Characteristics	ZNF326 expression		Total	*P* value
	Low	High		
Gender				0.779
Female	22	40	62	
Male	25	41	66	
Age				0.012
<50	27	28	55	
≧50	20	53	73	
WHO Grade				0.000
I	5	0	5	
II	23	20	43	
III	24	44	68	
IV	0	17	17	
Tumor location				0.852
Fronta	20	31	51	
Parietal	2	7	9	
Occipital	3	5	8	
Temporal	14	21	35	
Unknown	8	17	25	

Interestingly, we found that the expression of HDAC7 in NHA cell lines was also significantly lower than that in other glioma cell lines, similar to ZNF326 expression (Fig. 1d). Consistently, compared with the ZNF326 IHC staining results, the expression of HDAC7 was positively correlated with the expression of ZNF326 in gliomas (Additional file 2: Figure S1, Additional file 3: Table S1).

### ZNF326 promotes proliferation and invasion of glioma cells in vitro

**Fig. 2:**
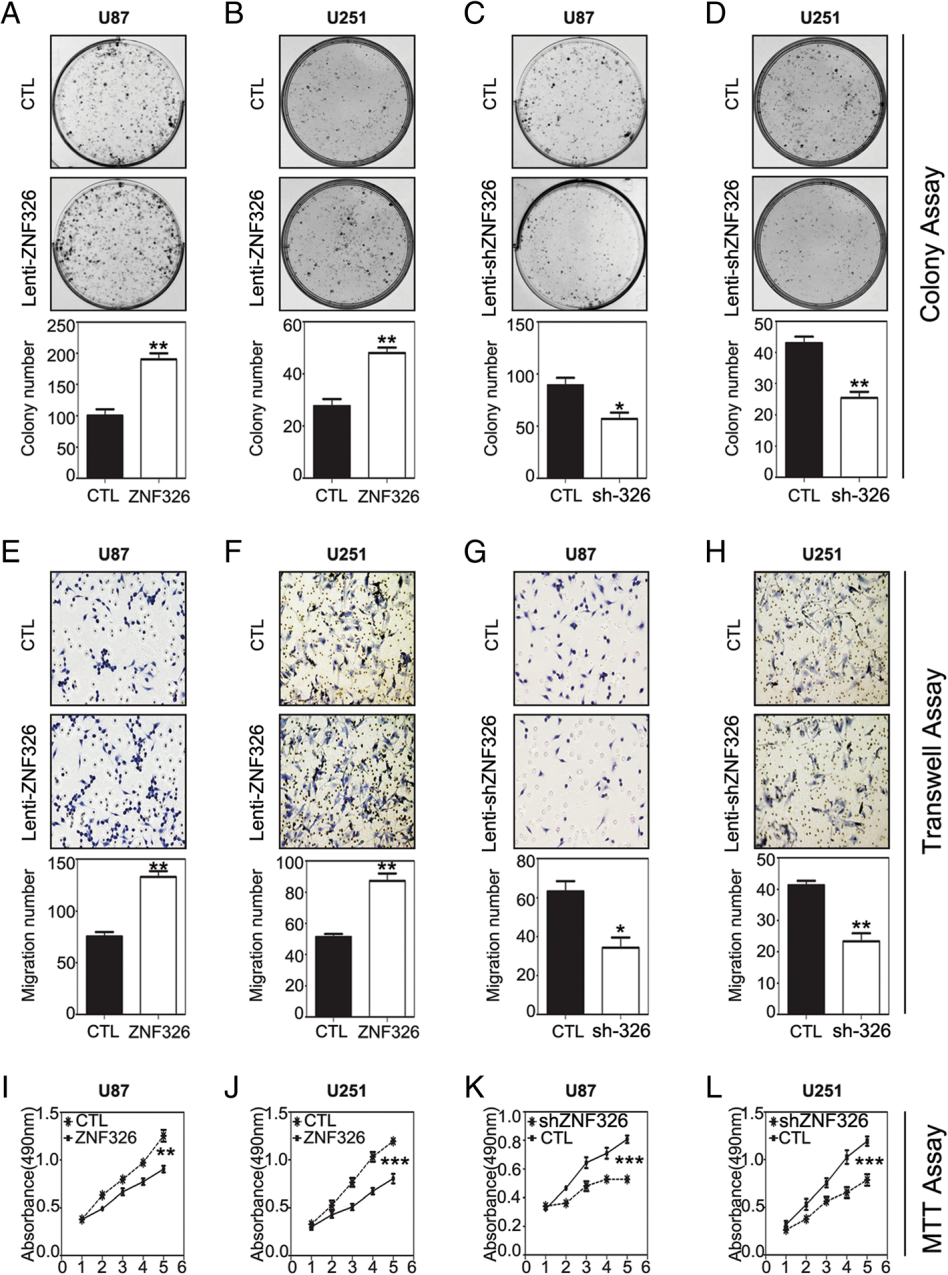
Impact of ZNF326 expression on the proliferation and invasiveness of glioma cells in vitro*.* ZNF326 overexpression significantly enhanced the colony formation (**a**, **b**), invasiveness (**e**, **f**, magnification-400×), and proliferation (**i**, **j**) of U87 and U251 glioma cell lines. Conversely, ZNF326 knockdown significantly inhibited colony formation (**c**, **d**), invasiveness (**g**, **h**, magnification-400×), and proliferation (**k**, **l**) of U87 and U251 glioma cell lines. CTL: control group. Each experiment was performed in triplicate. Columns: mean numbers. Bars: S.D. (*: *P* < 0.05; **: *P* < 0.01; ***: *P* < 0.001)

To verify whether ZNF326 has a causal role in regulating glioma cell phenotypes, we stably overexpressed ZNF326, using a lentivirus vector–based ZNF326 plasmid, in U87 and U251 cell lines. Compared with that in the control group, ZNF326 overexpression enhanced the following in U87 and U251 cells: colony formation (U87: CTL vs ZNF326, 100 ± 11 vs 190 ± 10, *P* < 0.01; U251: CTL vs ZNF326, 26 ± 3 vs 48 ± 2, *P* < 0.01; Fig. 2a-b), invasiveness (U87: CTL vs ZNF326, 75 ± 5 vs 132 ± 6, *P* < 0.01; U251: CTL vs ZNF326, 51 ± 2 vs 87 ± 5, *P* < 0.001; Fig. 2e-f). and proliferation (MTT assay; U87: CTL vs ZNF326, 0.904 ± 0.035 vs 1.254 ± 0.062, *P* < 0.01; U251: CTL vs ZNF326, 0.804 ± 0.049 vs 1.194 ± 0.032, *P* < 0.001; Fig. 2i-j), Conversely, ZNF326 knockdown in U87 and U251 cells by a lentivirus vector–based ZNF326 shRNA technique significantly weakened the following in both cell lines: colony formation (U87: CTL vs siZNF326, 89 ± 7 vs 57 ± 6, *P* < 0.05; U251: CTL vs siZNF326, 43 ± 2 vs 25 ± 2, *P* < 0.01, Fig. 2c-d), invasiveness (U87: CTL vs siZNF326, 63 ± 5 vs 34 ± 6, *P* < 0.05; U251: CTL vs siZNF326, 41 ± 2 vs 23 ± 3, *P* < 0.01; Fig. 2g-h) and proliferation (U87: CTL vs siZNF326, 0.808 ± 0.026 vs 0.528 ± 0.028, *p* < 0.001; U251: CTL vs siZNF326, 1.028 ± 0.050 vs 0.786 ± 0.061, *p* < 0.05; Fig. 2k-l).

### ZNF326 positively regulates the Wnt signalling pathway

**Fig. 3:**
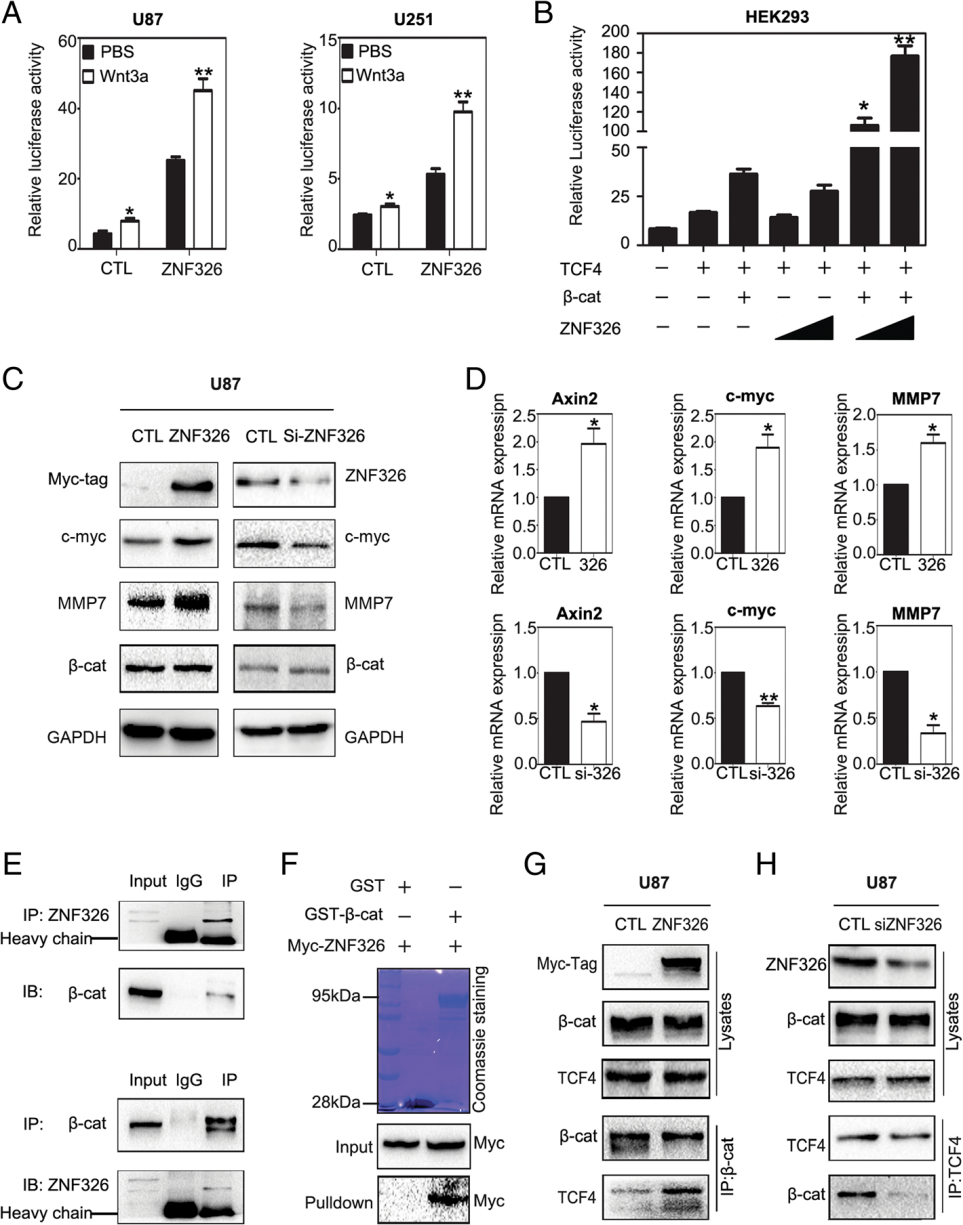
ZNF326 is a positive regulator of the Wnt pathway. **a**, **b** ZNF326 overexpression activates Wnt signalling. The Wnt TOPflash reporter was measured in U87 and U251 cells transfected with ZNF326 plasmid and an empty vector (negative control, CTL) and subsequently treated with control or Wnt3A-conditioned media for 6 h (**a**). On the basis of transfection of β-catenin and TCF4 in HEK293, TOPflash activity was significantly up-regulated after transfection of ZNF326 in a dose-dependent manner. Cells were treated with Wnt3A-conditioned media for 6 h (**b**). **c**, **d** The expression of ZNF326 was up-regulated or knocked down in U87 cell line; 48 h later, cells were lysed, Wnt-related genes and total β-catenin expression subsequently were assessed by immunoblotting (**c**) and RT-qPCR assays (**d**). GAPDH serves as a loading control. **e**, **f** ZNF326 directly interacts with β-catenin. Cell lysates from U87 cells were subjected to immunoprecipitation with anti-ZNF326 or anti-β-catenin antibody, and control IgG; precipitates were analysed by immunoblotting (**e**) In vitro. Purified Myc-ZNF326 and either GST or GST-β-catenin were incubated with glutathione sepharose for 6 h; precipitates were analysed by immunoblotting (**f**). **g**, **h** Endogenous ZNF326 enhanced the β-catenin-TCF4 combination. U87 cells were transfected with ZNF326 plasmid (**g**) or siRNA-ZNF326 (**h**). Relative interaction amount between β-catenin and TCF4 were detected by immunoprecipitation. Columns: mean numbers. Bars: S.D. (*: *P* < 0.05; **: *P* < 0.01)

The close association that has been reported between Wnt/β-catenin signalling and glioma tumorigenesis, combined with our KEGG analysis, predicted that ZNF326 is closely related to the Wnt/β-catenin pathway (Additional file 2: Figure S2-A). To verify this prediction, we first assessed the effect of ZNF326 on the activities of the Wnt pathway in glioma cell lines, using luciferase reporter assays. To readily detect differences, we pre-stimulated the Wnt signalling pathway using Wnt-3a [21]. In U87 and U251 cells, ectopic ZNF326 expression significantly increased the TOPflash activity of Wnt signalling induced by Wnt3a (Fig. 3a), and the Wnt pathway activity was gradually increased in a dose-dependent manner upon ZNF326 transfection in HEK293 cells (Fig. 3b).

ZNF326 overexpression in the U87 cell line significantly upregulated MMP7, C-myc and AXIN2 at both the protein and mRNA levels. Conversely, ZNF326 knockdown in U87 cells downregulated the expression of Wnt-related genes (Fig. 3c, d). This is consistent with the positive correlation between ZNF326 and Wnt target genes obtained by online analysis at the GEPIA website (http://gepia.cancer-pku.cn/, Additional file 2: Figure S2-B). In particular, we have noticed that the changes of ZNF326 expression have no significant effect on total β-catenin expression level. Notably, endogenous interaction between ZNF326 and β-catenin was detected in the U87 cell line (Fig. 3e) and in vitro glutathione S-transferase pull-down assays (Fig. 3f) confirmed their direct interaction. We next tested whether ZNF326 can promote TCF4–β-catenin interaction. Immunoprecipitation indicated that ZNF326 overexpression increased the interaction between β-catenin and TCF4 (Fig. 3g), whereas ZNF326 silencing decreased this interaction (Fig. 3).

### ZNF326 regulates target-gene HDAC7 expression through its transcriptional activation domain and zinc-finger structure

**Fig. 4:**
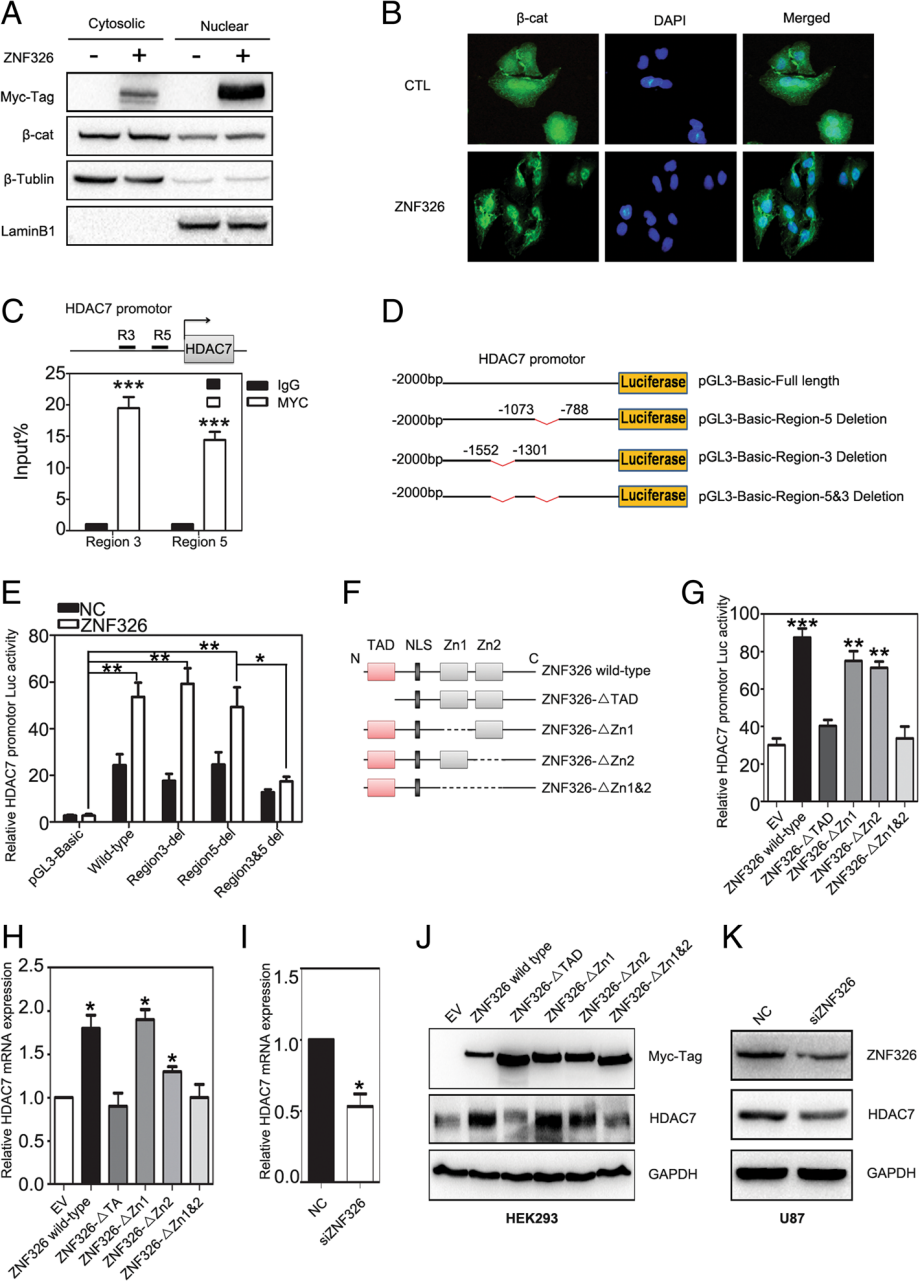
ZNF326 up-regulates HDAC7 expression at the transcriptional level. **a-c** After transfection of ZNF326 in HEK293 cells, the level of β-catenin nuclear import was assessed by cytosolic fractionation assay (**a**) and immunofluorescence assay (**b**), β-Tublin and LaminB1 were the loading controls for cytoplasm and nucleus, respectively. Chromatin was immunoprecipitated with the Myc-tag antibody or control IgG after transfection of Myc-ZNF326 plasmid in U87, followed by qPCR using primer pairs spanning the human HDAC7 promotor. Results are presented as percentage immunoprecipitated over input and are representative of three independent experiments (**c**). **e-f** Information about luciferase reporter plasmids of *HDAC7* promoter region plasmid (**d**) and the domain structure of ZNF326 (**f**). ZNF326 could promote the wild-type luciferase reporter activity of *HDAC7* promoter, and had no effect on the mutants. HEK293 cells were co-transfected with ZNF326 and HDAC7 promotor-luciferase reporters. Renilla luciferase served as a control for signal normalisation (**e**). **g** Transcriptional activation domain (TAD) and zinc-finger structure are essential for ZNF326 to promote HDAC7 transcription. HDAC7 transcriptional activity was measured by luciferase report assay after transfection of HDAC7 promotor-luciferase reporter plasmid and ZNF326 wild-type, as well as mutants in HEK293 cells**.** Data from a representative experiment are plotted as the mean of three replicates plus the standard deviation. **h-k** In the HEK293 cell line, HDAC7 mRNA and protein levels were detected by qPCR (**h**) and immunoblotting (**j**) after transfection of ZNF326 wild-type and mutants. After transfection of siRNA-ZNF326 in U87 cells, the mRNA and protein levels of HDAC7 were significantly downregulated (**i**, **k**). GAPDH served as a loading control. Columns: mean numbers. Bars: S.D. (*: *P* < 0.05; **: *P* < 0.01; ***: *P* < 0.001)

Interestingly, ZNF326 overexpression in HEK293 cells promotes β-catenin nuclear import, as shown using cytosolic fractionation assay and immunofluorescence (Fig. 4a-b). Next we investigated the underlying mechanism in this process. We transfected ZNF326 plasmid with MYC tag into the H1299 cell line with high transfection efficiency and searched for the potential target genes of ZNF326, using ChIP-seq assay. From the Broad-Peak model analysis, eight potential target genes were found to be associated with these promoter regions [22], including *HDAC7*, which was directly related to Wnt pathway activation [23, 24].

To study the phenomenon further, we designed eight pairs of qPCR primers for the *HDAC7* promoter region (0 to 2000 bp), and then transfected U87 cells with the Myc-tagged ZNF326 plasmid. ChIP-qPCR assay indicated that ZNF326, but not the control protein IgG, was bound to the promoter region of *HDAC7*, corresponding to primers no. 3 (− 1552 bp to − 1301 bp) and primers no. 5 (− 1073 bp to − 788 bp) (Fig. 4c). The *HDAC7* promoter was examined to identify the region critical to its activity and responsiveness to ZNF326 expression. Truncated reporters were constructed as shown in Fig. 4d. Luciferase reporter assay was performed to show that the ZNF326 regulated HDAC7 transcription activity by associating with the − 1552 to − 1301-bp and − 1073 to − 788-bp regions of the *HDAC7* promoter (Fig. 4e). Additionally, we explored the domains of ZNF326 that contribute to the binding with the *HDAC7* promoter. We designed a series of ZNF326 mutant plasmids, including the deletion of TAD region, single deletion of a zinc-finger structure, and complete deletion of both zinc-finger structures (Fig. 4f). HEK239 cells were then co-transfected with *HDAC7* promoter-reporter construct and these ZNF326 mutant plasmids. The results indicated that upon deletion of TAD and complete deletion of both zinc-finger structures, the mutant ZNF326 could not activate the *HDAC7* promoter-luciferase reporter activity (Fig. 4g). Consistently, RT-qPCR (Fig. 4h) and western blotting (Fig. 4j) also demonstrated that the deletion of the TAD region and complete deletion of the two zinc-finger structures in ZNF326 prevented the increase in mRNA and protein levels of HDAC7. Similarly, HDAC7 expression was downregulated by ZNF326 knockdown (Fig. 4i, k). Furthermore, the online analysis at the GEPIA website (http://gepia.cancer-pku.cn/) revealed that ZNF326 was positively correlated with HDAC7 expression (Additional file 2: Figure S3).

### HDAC7 deacetylates β-catenin at Lys49 and promotes β-catenin nuclear import

**Fig. 5:**
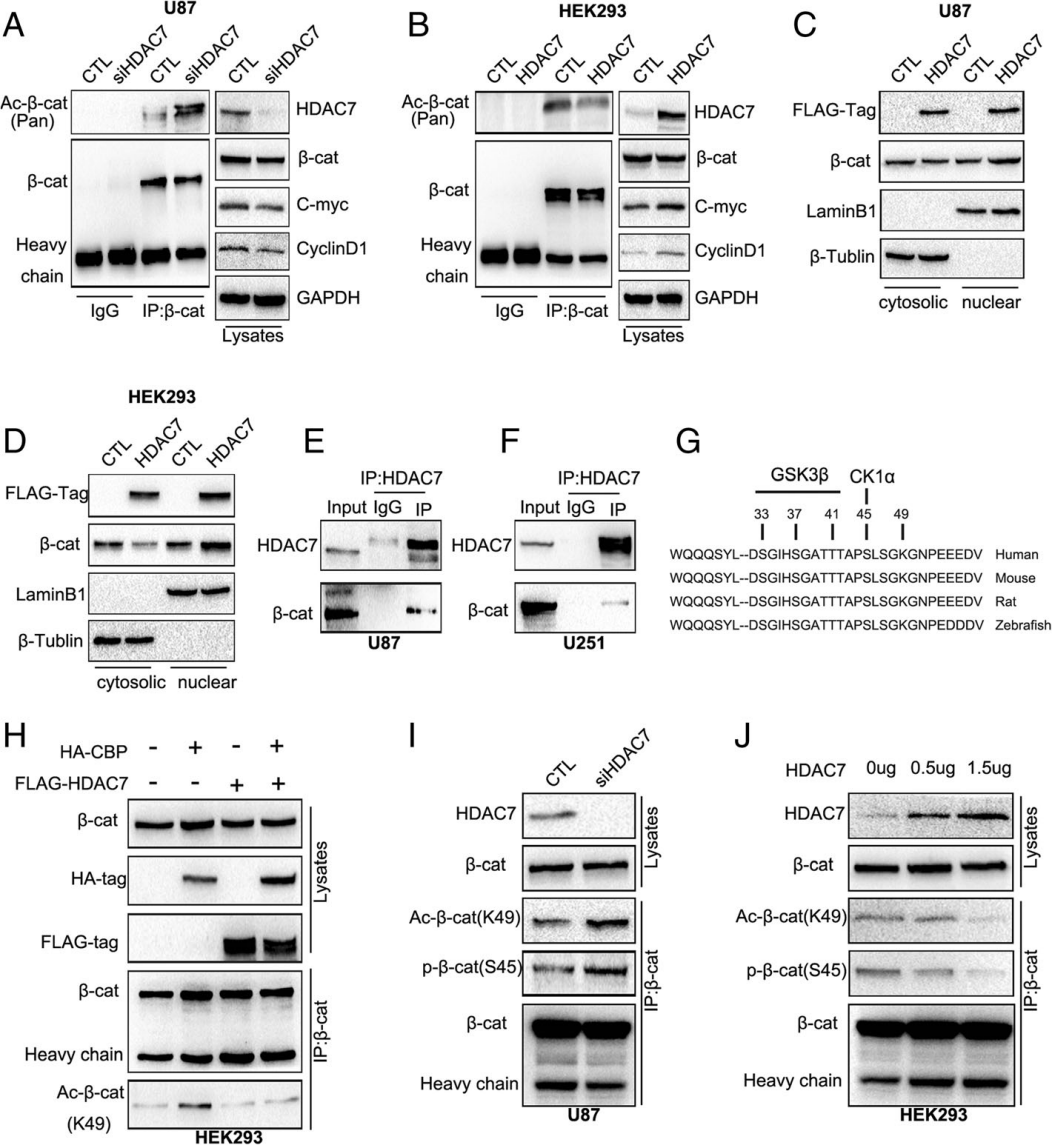
HDAC7 down-regulates the levels of β-catenin acetylation at Lys49 and phosphorylation at Ser45. **a**, **b** After transfection of siRNA-HDAC7 in U87 cells, the total acetylation level of β-catenin were upregulated and Wnt pathway target gene protein expression were downregulated, detected by immunoprecipitation and immunoblotting (**a**). But after transfection of HDAC plasmid with HEK293, the opposite result was obtained (**b**). No significant changes in total β-catenin protein were observed when both overexpression and knockdown of HDAC7. GAPDH served as a loading control. **c**, **d** Cytosolic fractionation and immunofluorescence assays were applied to detect β-catenin nuclear import after transfection of HDAC7 plasmid in U87 (**c**) or HEK293 (**d**) cells, indicating transfection of HDAC7 promotes the β-catenin nuclear import. β-Tublin and LaminB1 were cytoplasmic and nucleus loading controls, respectively. **e**, **f** Interaction between endogenous HDAC7 and β-catenin in U87 and U251 glioma cells. Cell lysates from U87 (**e**) and U251 (**f**) cells were subjected to immunoprecipitation with anti-HDAC7 antibody or control IgG and then examined for β-catenin expression by anti-β-catenin immunoblotting. **g**, **h** HDAC7 could reverse the up-regulation of β-catenin acetylation at Lys49 induced by CBP. Schematic diagram of β-catenin serine/threonine and lysine residues (**g**). The HA-CBP and FLAG-HDAC7 plasmids were co-transfected in HEK293 cells. Immunoprecipitation and immunoblotting assays were used to detect the acetylation level of β-catenin at Lys49. total β-catenin was the loading control (H). **i**, **j** After knockdown of HDAC7 in U87 cells, β-catenin acetylation level at Lys49 and phosphorylation level at Ser45 were significantly upregulated (**i**), With the increase of HDAC7 plasmid concentration, the level of β-catenin acetylation and phosphorylation gradually decreased (**j**). the total β-catenin level was the loading control

We further examined the role of HDAC7 in the Wnt pathway. We found that HDAC7 knockdown in the U87 cell line, using siRNA-HDAC7, significantly decreased Wnt target genes, C-myc and MMP7 expression, which was in accordance with the positive correlation between HDAC7 and some other Wnt target genes (such as *CD44, PTGS2,* and *MMP2*) revealed by GEPIA online analysis (Additional file 2: Figure S4). Meanwhile, the total acetylation level of β-catenin was also upregulated (Fig. 5a). Conversely, HDAC7 ectopic expression in HEK293 cells increased the expression of Wnt-related genes and decreased β-catenin acetylation level (Fig. 5b). Notably, HDAC7 overexpression promoted the nuclear import of β-catenin (Fig. 5c-d).

Based on the experimental data above, we hypothesised that HDAC7 regulates the level of β-catenin nuclear import by affecting its acetylation level. To test this, immunoprecipitation assay in U87 and U251 cells was conducted to demonstrate that HDAC7 could interact with β-catenin (Fig. 5e-f). According to the literature that states that HDAC6 can deacetylate β-catenin at Lys49 [25], we tested whether HDAC7 also has a similar effect. We thus co-transfected HDAC7 and HA-CBP (CREB binding protein, a protein that can increase β-catenin acetylation at Lys-49) plasmids in the HEK293 cell line [25]. The results showed that HDAC7 could also reverse the up-regulation of β-catenin acetylation at Lys49 induced by CBP (Fig. 5g, h), whereas HDAC7 silencing in U87 cells significantly increased β-catenin acetylation at Lys49 (Fig. 5i). CK1α-mediated phosphorylation of β-catenin at the serine 45 residue is a key step for β-catenin degradation [26]. Our results showed that HDAC7 knockdown significantly upregulated the level of phosphorylation of β-catenin Ser45 (Fig. 5i). Accordingly, the levels of β-catenin acetylation at Lys49 and β-catenin phosphorylation at Ser45 were significantly decreased in a dose-dependent manner after HDAC7 overexpression in HEK293 cells (Fig. 5j).

### ZNF 326 promotes β-catenin nuclear import by increasing HDAC7 expression

**Fig. 6:**
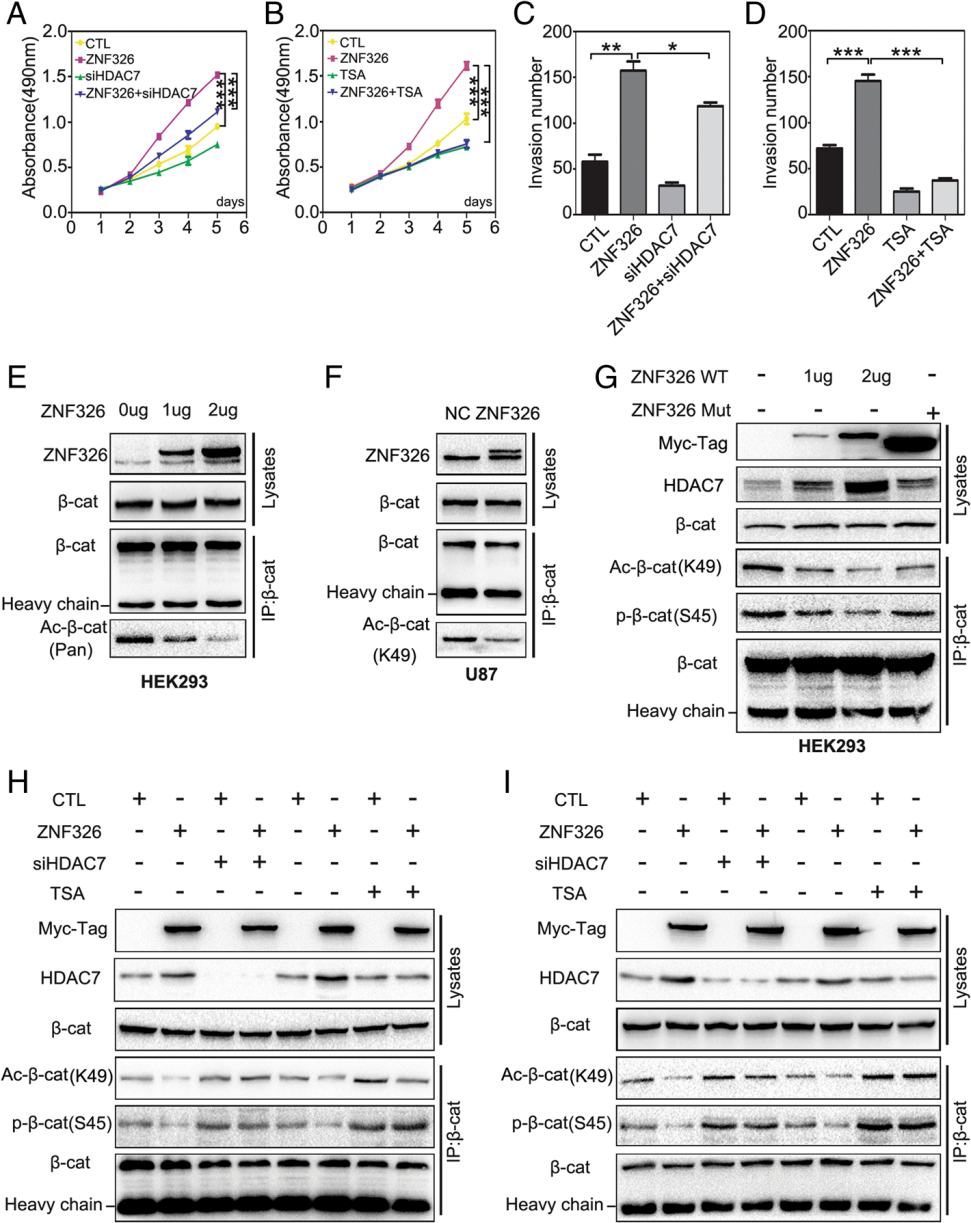
ZNF326 down-regulates acetylation of β-catenin at Lys49 and phosphorylation at Ser45 through increasing HDAC7 expression. **a-d** ZNF326 and siRNA-HDAC7 were co-transfected into U87, or the inhibitor TSA (10 nM) was added in U87 cells. MTT and Transwell assays were applied to detect the decreased proliferation (**a**, **b**) and invasiveness (**c**, **d**). Columns: mean numbers. Bars: S.D. (*: *P* < 0.05; **: *P* < 0.01; ***: *P* < 0.001). **e**, **f** After transfection of the ZNF326 plasmid in the HEK293 cell line, immunoprecipitation and immunoblotting showed a significant decrease in the level of total β-catenin acetylation in a dose-dependent manner (**e**). ZNF326 overexpression in U87 also significantly decreased β-catenin acetylation at Lys49 (**f**); total β-catenin served as a loading control. **g** Wild-type ZNF326 deregulated the acetylation level at Lys49 and the phosphorylation level at Ser45 of β-catenin, while the mutant (both zinc-finger structures deleted) abrogated this effect; total β-catenin served as a loading control. **h**, **i** Co-transfection of ZNF326 and siRNA-HDAC7 or TSA added (10 nM) in U87 (**h**) and U251 cells (**i**), the downregulation of β-catenin acetylation level at Lys49 and phosphorylation level at Ser45 induced by ZNF326 were abrogated; total β-catenin served as the loading control

To further understand HDAC7 regulation by ZNF326, siRNA-HDAC7 or TSA were used to knock out HDAC7 expression or inhibit HDAC7 activity, respectively. The proliferation and invasiveness of glioma cells were reversed by HDAC7 silencing or HDAC7 inhibition, as confirmed by MTT assay and Transwell assay, respectively (Fig. 6a-d, Additional file 2: Figure S5). On the other hand, immunoprecipitation assay demonstrated that ZNF326 overexpression in HEK293 clearly decreased the total β-catenin acetylation level in a dose-dependent manner (Fig. 6e). Overexpression of ZNF326 in U87 cells also deacetylated β-catenin at Lys49 (Fig. 6f). Furthermore, we transfected ZNF326 wild-type and ZNF326 mutant plasmid lacking both the zinc-finger structures and the TAD domain in HEK293. The results showed that wild-type ZNF326 could increase the level of HDAC7 protein, and reduce the level of β-catenin acetylation at Lys49 and phosphorylation at Ser45, and that the mutant abrogated this effect (Fig. 6g). Finally, after overexpression of mutant ZNF326 or knocking down HDAC7 or adding TSA in U87 and U251 cells, the effect of ZNF326 on the reduction of β-catenin acetylation at Lys49 and phosphorylation at Ser45 disappeared (Fig. 6h, i).

### ZNF326 promotes tumour growth in xenograft model of nude mice in vivo

**Fig. 7:**
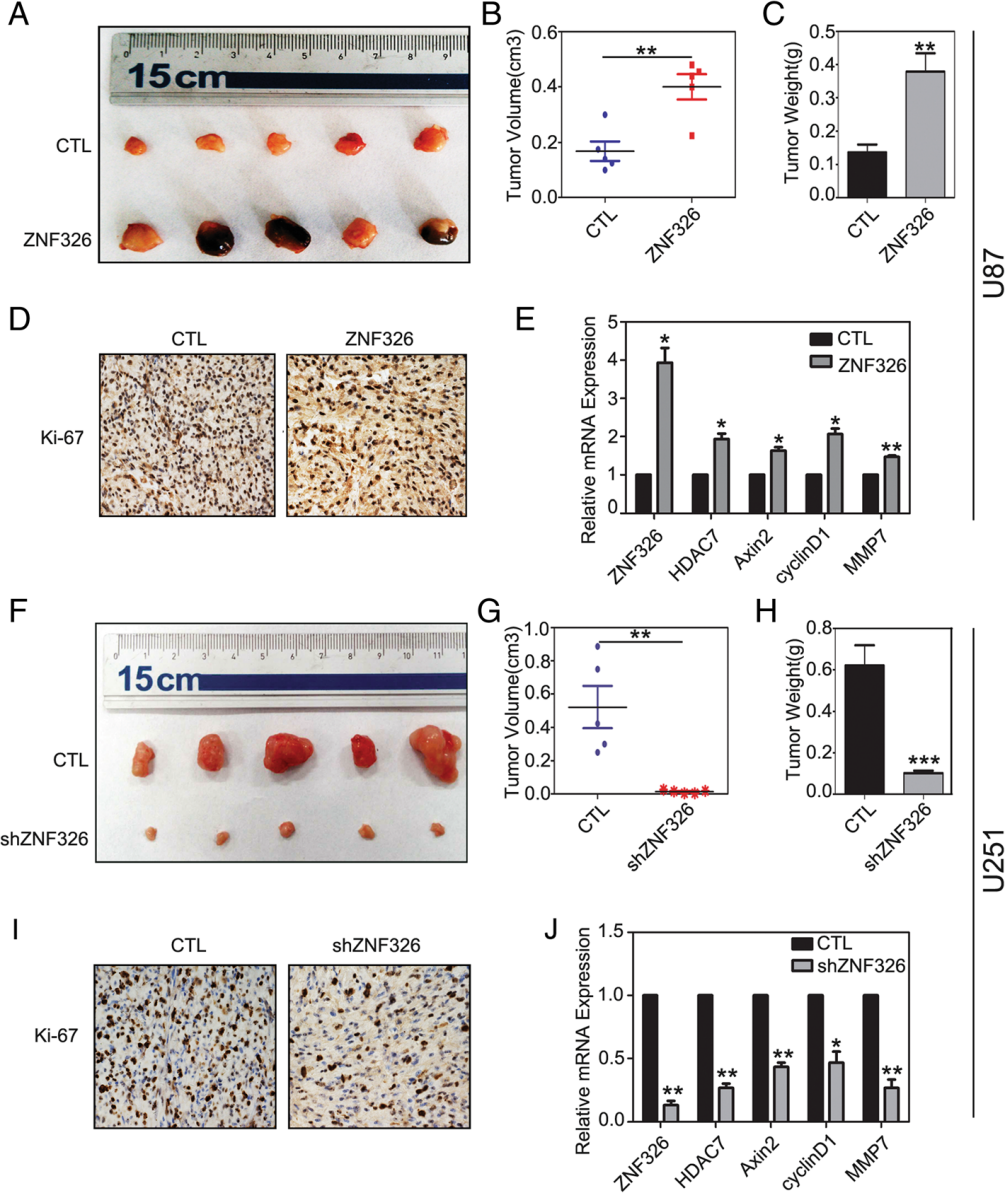
ZNF326 promotes the tumour xenografts formation in nude mice. ZNF326 promotes glioma tumorigenesis. The subcutaneous injection of U87 cells stably expressing ZNF326 (puromycin screening) into nude mice (*n* = 5) significantly accelerated tumor formation compared with the control group (*n* = 5) (**a**, **b**, **c**). At the same time, the Ki-67 index (nuclear staining/HPF, CTL vs. ZNF326; 89 ± 9 vs. 178 ± 16, *P* < 0.05, **d**) and the downstream target gene expression of Wnt pathway and HDAC7 (**e**) significantly increased. In contrast, the injection of U251 cells transfected with lenti-shRNA-ZNF326 (puromycin screening) attenuated tumor formation (**f-h**), Ki-67 index (nuclear staining/HPF, CTL vs. shZNF326; 76 ± 6 vs. 33 ± 4, *P* < 0.05, **i**), as well as the Wnt target genes (**j**). Statistical significance was determined by a two-tailed, unpaired *t*-test. Columns: mean numbers. Bars: S.D. (*: *P* < 0.05; **: *P* < 0.01; ***: *P* < 0.001)

To verify the impact of ZNF326 on the tumour growth of glioma cells in vivo, we evaluated the role of ZNF326 in tumour formation of U87 and U251 cells, using a xenograft model of nude mice. As shown in Fig. 7a-c, stable expression of ZNF326 using lentivirus in U87 cells (selected with puromycin, 5 μg/mL) significantly promoted tumour growth in vivo when compared with that in the control group (CTL vs ZNF326, volume:0.168 ± 0.035 vs 0.400 ± 0.046, *P* < 0.01; weight:0.136 ± 0.024 vs 0.378 ± 0.056, *P* < 0.01). Consistent with this observation, Ki-67 staining, and transcription of Wnt target genes (*AXIN2*, *CCND1*, and *MMP7*) and HDAC7 were markedly enhanced in tumours in which ZNF326 was overexpressed (Fig. 7d-e). In addition, we used the lentivirus-shZNF326 to knock down ZNF326 (selected with puromycin, 10 μg/mL) in the U251 cell line. The results showed that, the volume and weight of xenografts in nude mice after ZNF326 knockdown significantly decreased (CTL vs shZNF326, volume: 0.522 + 0.126 vs 0.013 + 0.004, *P* < 0.01; weight: 0.620 + 0.097 vs 0.101 + 0.012, *P* < 0.001, Fig. 7f-h); and the Ki-67 staining and mRNA levels of Wnt pathway target genes and HDAC7 decreased (Fig. 7i-j). These results suggested that ZNF326 promotes tumor growth by up-regulating the expression of HDAC7 and activating Wnt pathway.

**Fig. 8:**
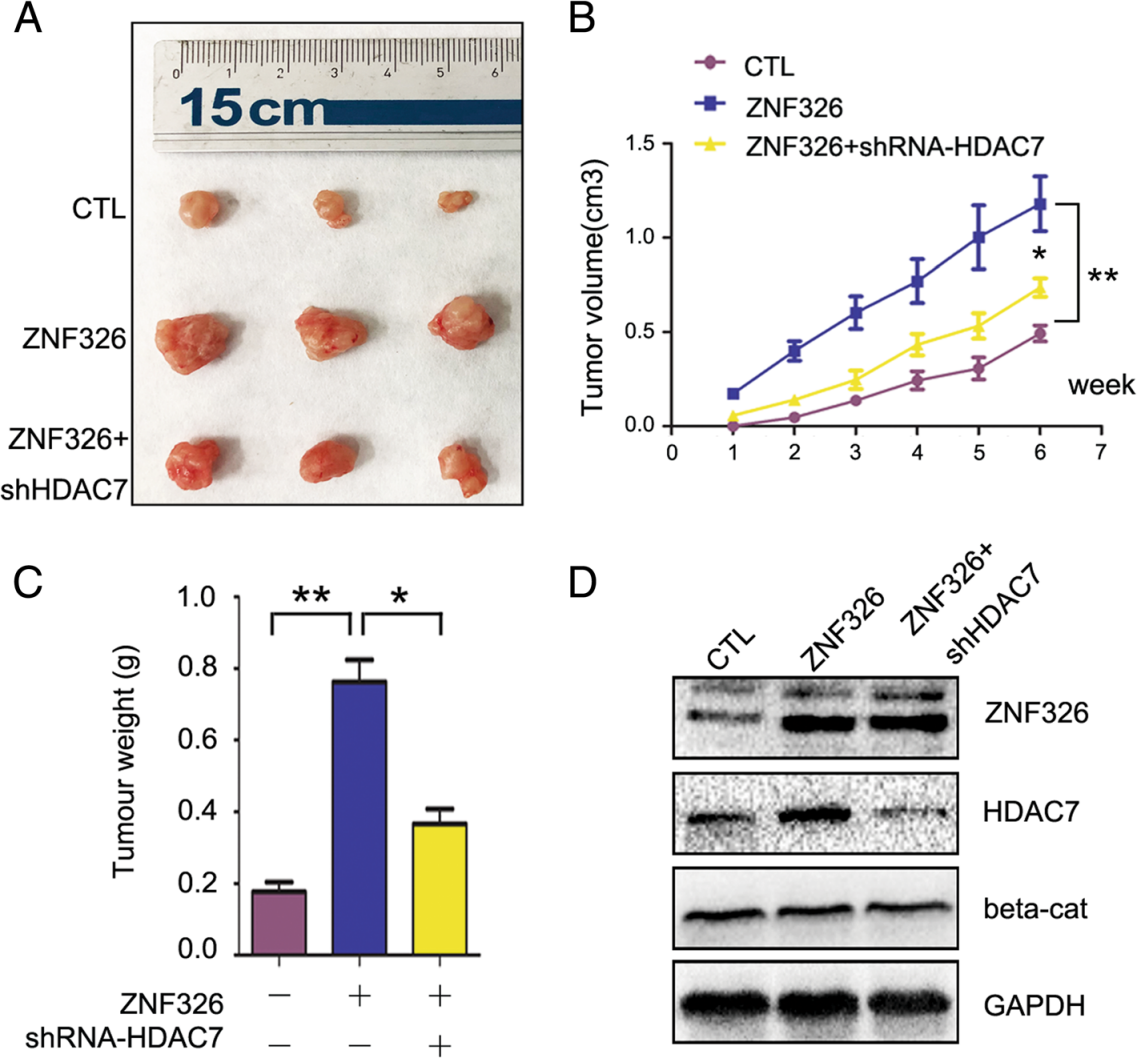
ZNF326 promotes tumor growth, at least partially through HDAC7. **a-c** ZNF326 and shRNA-HDAC7 were co-transfected in U87 cells, and exnografts formation assay in nude mice was performed to detect the changes in the tumor growth. Statistical significance was determined by a two-tailed, unpaired *t*-test. Columns: mean numbers. Bars: S.D. (*: *P* < 0.05; **: *P* < 0.01). **b** Transfection efficiency of ZNF326 and HDAC7, as well as β-catenin expression exnografts were detected using immunoblotting. GAPDH was used as a loading control

To confirm the hypothesis above, we attempted to compare the effects of ZNF326 and ZNF326 + shRNA-HDAC7 on tumour growth. We found the volume and weight of xenografts in ZNF326 + shRNA-HDAC7 group markedly reduced compared to the ZNF326 group (ZNF326 vs ZNF326 + shRNA-HDAC7, volume: 1.180 + 0.141 vs 0.736 + 0.052, *P* < 0.05; weight: 0.763 + 0.060 vs 0.366 + 0.041, *P* < 0.05), indicating that ZNF326 promotes tumour growth, at least partially through HDAC7 (Fig. 8a-c). In particular, we noticed transfection of ZNF326 or shRNA-HDAC7 had no significant effect on the total amount of β-catenin in xenografts tissue (Fig. 8d), which was consistent with the experimental results in vitro.

## Discussion

The zinc-finger protein ZNF326 was initially found by Lee et al. in the NIH3T3 cell line, and detected to be highly expressed in the brain and neural tube of the E11.5 embryo, which indicated that ZNF326 might play an important role in the process of nerve development [19, 20]. Recently, it was reported that ZNF326 may form a DBIRD complex with DBC1in human embryonic kidney cell line HEK293 through its S1RNA domain. This complex acts on mRNA particles and RNAII (polymerase), which regulates gene transcription and alternative splicing [27].These led us to explore the underlying role of ZNF326 in the development and progression of glioma. In this study, we uncovered that ZNF326 expression is upregulated in glioma specimens, which is also consistent with the result obtained from the TCGA database. Statistical analysis indicated high ZNF326 expression positively correlated with tumour grade, which hinted that ZNF326 might function as an oncogene in glioma. In vitro and in vivo experiments verified that ZNF326 can promote glioma cell proliferation and invasiveness and tumour xenograft formation in nude mice. These results were sufficient to conclude that ZNF326 is a tumour-promoting factor in glioma.

So far, the underlying mechanism of ZNF326 on gliomagenesis is almost unknown. Mounting evidence shows that overstimulation of Wnt signalling can lead to malignant progression of glioma. We found ZNF326 transfection in glioma cells significantly increased the activity of the Wnt signalling pathway and its target gene expression, and there was an opposite effect upon ZNF326 knockdown. This is consistent with the positive correlation between ZNF326 and Wnt target genes as obtained from our GEPIA website (http://gepia.cancer-pku.cn/) and gene enrichment analysis. After fully determining the relationship between ZNF326 and the Wnt signalling pathway, we focused on the role of ZNF326, which is multifaceted. On one hand, ZNF326 can directly bind with β-catenin in the nucleus. This combination can further promote the binding of β-catenin and transcription factor TCF4, thus activating the transcription of target genes of the Wnt pathway. ZNF326 functions as a co-activator in this process. Unfortunately, we are not clear about the molecular structural basis of the combination of ZNF326 and β-catenin, and will explore it in our future research.

On the other hand, ZNF326 can also play a vital role as a transcription factor. We used ChIP-seq and ChIP-qPCR to screen and prove that HDAC7 is one of the target genes downstream to ZNF326. Moreover, ZNF326 transfection can significantly up-regulate the transcriptional level of HDAC7. Subsequently, we further designed and constructed a series of ZNF326 mutant plasmids, and proved that wild-type ZNF326 can bind to the *HDAC7* promoter (− 1552 bp to − 1301 bp and − 1073 to − 788 bp) through its TAD and zinc-finger structures, whereas the mutant ZNF326, which cannot bind to the *HDAC7* promoter region, abrogates this effect. Thus, it was further confirmed that ZNF326 acts as a transcription factor to positively regulate the transcriptional activity of target gene *HDAC7*.

HDAC7 is a member of the HDAC family, and plays an important role in many biological processes and is closely related to the occurrence of cancer. Li et al reported that inactivation of HDAC6 increases the level of β-catenin acetylation at Lys49, which leads to increased EGF-induced β-catenin nucleation, and promotes the malignant phenotype of colon cancer cells [25]. We found that after transfection of ZNF326, the total β-catenin expression level was not significantly altered; so we focused our attention on the effect of HDAC7 on β–catenin function. Immunoprecipitation assay showed that the interaction between HDAC7 and β-catenin decreased the level of β-catenin acetylation at Lys49 and then downregulated phosphorylation at Ser45. Transfection of ZNF326 mutants that could not bind to the *HDAC7* promoter abrogated this effect. In addition, after overexpressing mutant ZNF326 and knocking down HDAC7 or adding TSA to inhibit the function of HDACs, the down-regulation effect of ZNF326 on β-catenin acetylation at Lys49 and phosphorylation at Ser45 disappeared. Thus, it is clear that the change in β-catenin acetylation and phosphorylation is achieved by ZNF326 through HDAC7. Our results support the fact that many non-histone proteins found in recent studies can also serve as substrates of HDACs to participate in many biological processes [28–30]. The high expression of HDACs is usually associated with progression of the tumour and poor prognosis of the patient [31–33]; the current HDAC inhibitors are also becoming a new class of anti-tumour drugs [34]. Notably, we found the glioma cells with co-transfecion of ZNF326 and shRNA-HDAC7 still exerted the higher tumor formation ability compared to the control group, or in vitro*,* transfection siRNA-HADC7 just partially abolished the promotion effect of ZNF326 in glioma proliferation, which indicates ZNF326 also could promotes glioma progression via HDAC7-independent manner. In our previous study, eg. *ERCC1*, a gene closely related to cell proliferation, is also the target gene of ZNF326 [22], If ZNF326 can promote the proliferation of glioma through both of them, HDAC7 knockdown alone cannot completely inhibit the growth of glioma cells induced by ZNF326. Therefore, which gene, such as *HDAC7*, *ERCC1*, *LTBP4* and *ZNF383*, could play a major role in glioma proliferation need further study and investigation.

So, is there any correlation between the effect of HDAC7 on the acetylation and phosphorylation of β-catenin? Why does HDAC7 inhibit the phosphorylation of β-catenin while the total level of β-catenin remains unchanged both in vitro and in vivo? HDAC7 interacts with β-catenin in the cytoplasm and decreases the acetylation level of β-catenin at Lys-49. Thereafter, the steric hindrance was changed and phosphorylation level with the adjacent 45th serine was inhibited. Therefore, up-regulation of HDAC7 expression can decrease the level of p-β-catenin, which is consistent with previous studies on HDAC6 [25]. In order to explain the decrease in p-β-catenin, the total level of β-catenin did not yet change significantly, we examined whether CK1α expression changes after dual-regulation of ZNF326 and HDAC7, because CK1α-mediated phosphorylation of β-catenin at the serine 45 residue is a key step for β-catenin degradation [26]. The results showed that CK1α did not change while ZNF326 and HDAC7 were changed (Additional file 2: Figure S6). HDAC7 inhibits the phosphorylation of β-catenin by inhibiting the acetylation of β-catenin, while non-phosphorylated β-catenin imports the nucleus and activates the Wnt pathway. To conclude, the inhibition of phosphorylation of β-catenin by HDAC7 only changed its cytosolic-nuclear redistribution.

In the initial stage of our experiment, we noticed that the opposite results were reported in three negative breast cancers (TNBC) by Rangel et al, who found that the expression of ZNF326 in TNBC specimens was decreased and ZNF326 overexpression was able to weaken the ability of the transplanted tumour formation [35], indicating ZNF326 acts as a tumor suppressor gene. In this regard, we analyzed the possible reasons: the previous experiments used rabbit polyclonal antibody against ZNF326, and there may be some differences between the polyclonal antibody and the monoclonal antibody we used. We also attempted to compare the possible differences between them, but the polyclonal antibody was not available. In addition, Madhumitha et al. also reported that PRMT5/WDR77 can regulate the expression of ZNF326 in the TNBC cell line MDA-MB-231 by regulating ZNF326 promotor methylation, which then regulates the expression of the downstream genes such as *REPIN1/AP4* and *ST3GAL6* [36]. These data suggest that ZNF326 may play different biological roles in tumours of different tissue types and different genetic backgrounds. It is not clear why ZNF326 is overexpressed in gliomas, whether it is due to gene amplification, regulation of upstream genes or impaired metabolism, and whether the high expression of ZNF326 is tissue-specific, etc. need further study and confirmation.

**Fig. 9:**
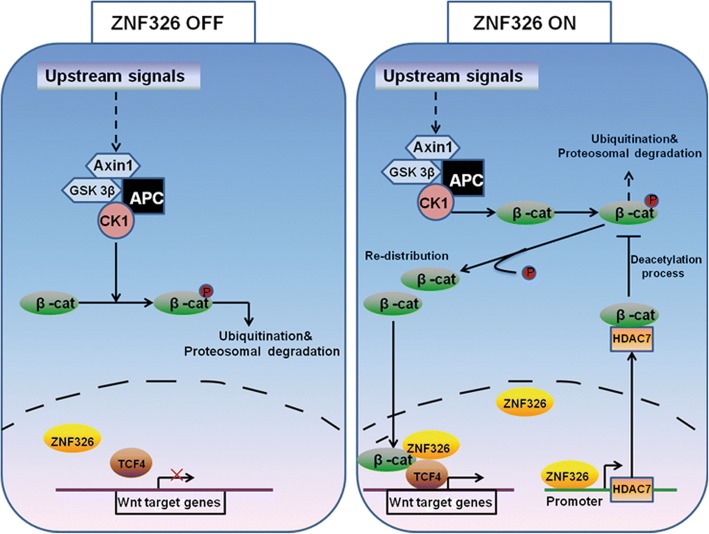
Schematic diagram of the molecular mechanism of ZNF326-mediated regulation of Wnt signalling

To conclude, ZNF326 expression is deregulated in human glioma, and its up-regulation is associated with tumour grade in patients with glioma. Both in vivo and in vitro experiments demonstrated that ZNF326 plays a role as a tumour-promoting factor through activation of the Wnt pathway. ZNF326, as a transcription activator, binds to the *HDAC7* promoter region and activates the transcription of HDAC7. HDAC7 interacts with β-catenin and reduces the level of β-catenin acetylation at Lys49, leading to the reduction of phosphorylation levels at Ser45. This leads to the nuclear accumulation of β-catenin and activates the Wnt signalling pathway. The β-catenin in the nucleus combines with ZNF326 and acts as a transcriptional co-activator of Wnt target genes (Fig. 9). Therefore, ZNF326-HDAC7-β-catenin forms a regulatory loop that activates and strengthens Wnt pathway activity, thereby promoting the malignant phenotype of glioma cells. These results not only reveal the role and mechanism of ZNF326 in carcinogenesis and glioma progression, but also suggest new targets for drug development and drug resistance research.

## Conclusions

In summary, our findings demonstrated the expression level of ZNF326 in glioma tissue was positively correlated with its grades. ZNF326 could activate *HDAC7* transcription via its transcriptional activation domain and zinc-finger structures. The interaction of the up-regulated HDAC7 with β-catenin led to β-catenin posttranscriptional modification and promoted its import into the nucleus, then activates the Wnt signalling pathway. On the other hand, ZNF326 directly associated with β-catenin in the nucleus, and enhanced the binding of β-catenin to TCF-4, serving as a co-activator in stimulating Wnt pathway. Therefore, ZNF326 promotes the malignant phenotype of human glioma via ZNF326-HDAC7-β-catenin signalling, which is one of its biological mechanisms.

## Additional files

Additional file 1: Materials and Methods (DOCX 22 kb)

Additional file 2: **Figure S1.** The positive correlation between ZNF326 and HDAC7 expressions in gliomas with different grade. (A, D, G, J): Representative images of glioma specimens using H&E staining were shown (Grade I-IV, Magnification 400×). IHC staining of ZNF326 and HDAC7 expressions in gliomas with different grades outlined below: (B, C): ZNF326 and HDAC7 were both negative expressed in pilocytic astrocytoma (grade I, Magnification 400×). (E, F): ZNF326 (<25%, +) and HDAC7 (<25% positive cells) were detected in diffuse astrocytoma (grade II, Magnification 400×). (H, I): ZNF326 was nuclear positive (nearly 50%, ++), while HDAC7 (>50% positive cells, ++) was perinuclear cytoplasm positive in anaplastic astrocytoma (grade III, Magnification 400×), (K, L): ZNF326 and HDAC7 were strongly expressed (nucleus and cytoplasm, respectively, >75% positive cells, +++) in glioblastoma with grade IV (Magnification 400×). **Figure S2.** ZNF326 expression is associated with expression of Wnt target genes. A: Bioinformatics KEGG test was used to analyse the correlation between ZNF326 and the Wnt pathway. B: Positive correlation between ZNF326 and the four common Wnt signalling pathway target genes in glioma, analysed at the GEPIA website. **Figure S3.** Positive correlation between ZNF326 and HDAC7 in glioma, analysed at the GEPIA website. **Figure S4.** Positive correlation between HDAC7 and Wnt signalling pathway target genes in glioma, analysed at the GEPIA website. **Figure S5.** ZNF326 and siRNA-HDAC7 were co-transfected, or TSA (10nM) was added in U87 cells, and Transwell assays were performed to detect the changes in the invasiveness of the glioma cells. **Figure S6.** (A-D): ZNF326, siRNA-ZNF326, HDAC7 and siRNA-HDAC7 were transfected in U87 cells, respectively, and immunoblotting assay was performed to detect the changes in the expression of β-catenin and CK1α. GAPDH was used as a loading control. (ZIP 12193 kb)

Additional file 3: **Table S1.** The correlation between the expression of ZNF326 and HDAC7 in glioma. (DOCX 14 kb)

## Abbreviations

ChIP: Chromatin Immunoprecipitation
CTL: Control
GAPDH: Glyceraldehyde-3-phosphate dehydrogenase
HDAC7: Histone deacetylase-7
MMP: Matrix metalloproteinase
RT-qPCR: Reverse transcription quantitative polymerase chain reaction
TSA: Trichostatin A
ZNF326: Zinc-finger protein-326
