# Correction to: ZNF326 promotes malignant phenotype of glioma by up-regulating HDAC7 expression and activating Wnt pathway

**DOI:** 10.1186/s13046-020-1526-z

**Published:** 2020-01-16

**Authors:** Xinmiao Yu, Minghao Wang, Jingjing Wu, Qiang Han, Xiupeng Zhang

**Affiliations:** 1 Department of Surgical Oncology and Breast Surgery, First Affiliated Hospital of China Medical University, Shenyang, China; 2 Department of Neurosurgery, First Affiliated Hospital of China Medical University, Shenyang, 110001 China; 3 Department of Pathology, First Affiliated Hospital of Fujian Medical University, Fuzhou, China; 4 Department of Pathology, College of Basic Medical Sciences, and First Affiliated Hospital of China Medical University, Shenyang, China

**Correction to: J Exp Clin Cancer Res (2019) 38:40**

**https://doi.org/10.1186/s13046-019-1031-4**

In the original publication of this manuscript [1], the author mislabeled the CTL group and ZNF326 group in Fig.2-I,J (MTT result). The revised Fig. 2 is shown below.

**Fig. 2:**
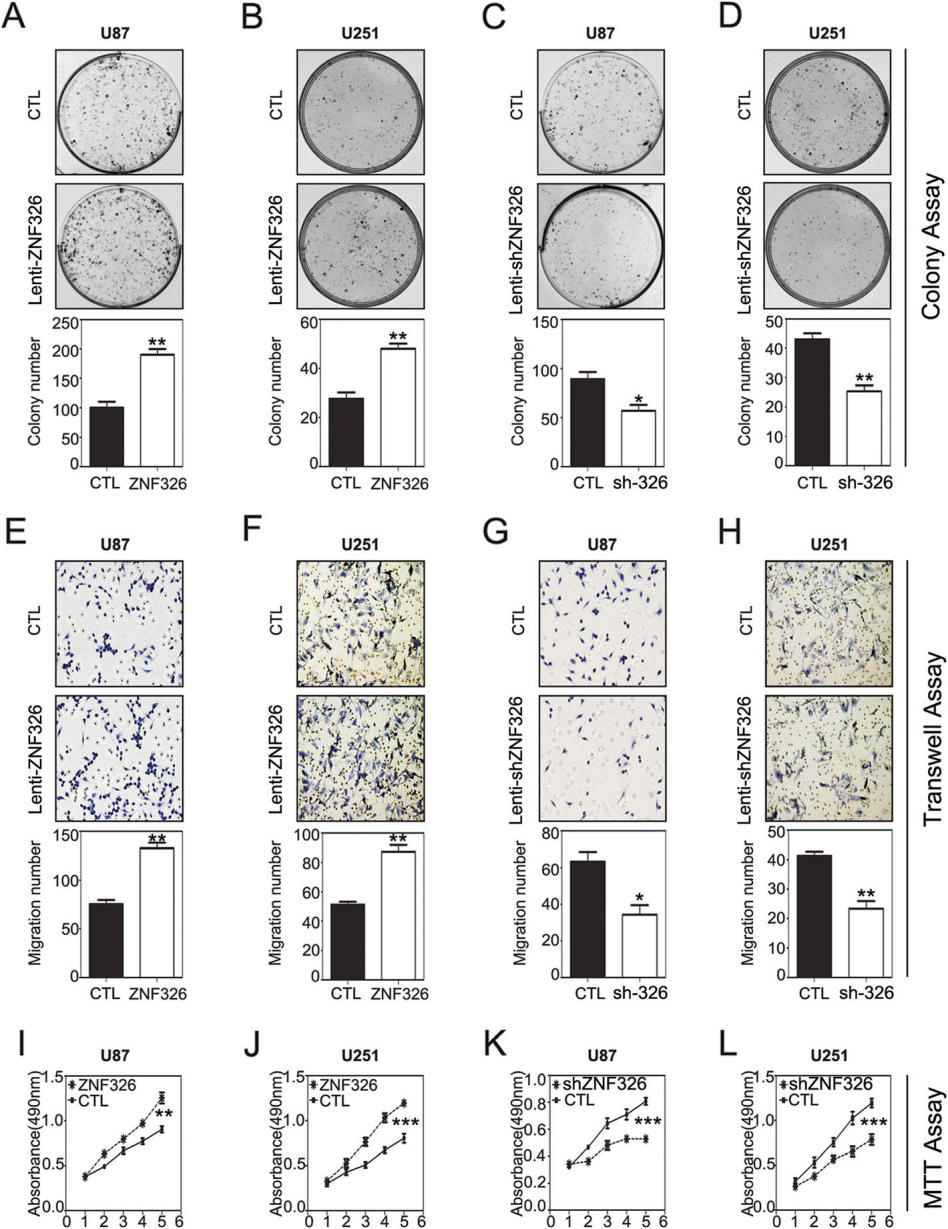
Impact of ZNF326 expression on the proliferation and invasiveness of glioma cells in vitro*.* ZNF326 overexpression significantly enhanced the colony formation (**a**, **b**), invasiveness (**e**, **f**, magnification-400×), and proliferation (**i**, **j**) of U87 and U251 glioma cell lines. Conversely, ZNF326 knockdown significantly inhibited colony formation (**c**, **d**), invasiveness (**g**, **h**, magnification-400×), and proliferation (**k**, **l**) of U87 and U251 glioma cell lines. CTL: control group. Each experiment was performed in triplicate. Columns: mean numbers. Bars: S.D. (*: *P* < 0.05; **: *P* < 0.01; ***: *P* < 0.001)

The authors sincerely apologize for the inconvenience caused to the readers.
