# Supplementary material for: ZNF326 promotes malignant phenotype of glioma by up-regulating HDAC7 expression and activating Wnt pathway
Source: J Exp Clin Cancer Res. 2019 Jan 28;38:40. doi: 10.1186/s13046-019-1031-4 (PMC6350303; doi:10.1186/s13046-019-1031-4)
Supplement: Supplementary file 1 — Materials and Methods (DOCX 22 kb) [file 13046_2019_1031_MOESM1_ESM.docx]

**MATERIALS AND METHODS**

**Western blot analysis and immunoprecipitation**

Immunoprecipitation (IP) and immunoblotting (IB) were carried out with antibodies against the following proteins: ZNF326 (sc-390606, 1:200/IB, 5 µg/IP), TCF4 (sc-166699, 1:200/IB, 5 µg/IP), HDAC7 (sc-74563, 1:200/IB, 5 µg/IP), and CK1α (sc-74582, 1:200/IB), GAPDH (sc-293335, 1:1000/IB), purchased from Santa Cruz Biotechnology Inc. (CA, USA). Myc-Tag (#2276, 1:1000/IB, 1:50/IP), FLAG-Tag (#14793, 1:1000/IB), HA-Tag (#3724, 1:1000/IB), C-myc (#9402, 1:1000/IB), MMP7 (#3801, 1:1000/IB), CyclinD1 (#2922, 1:1000/IB), Rabbit β-catenin (#8480, 1:1000/IB, 1:100/IP), acetyl-β-catenin (Lys49，#9534, 1:1000/IB), p-β-catenin (Ser45，#9564, 1:1000/IB), acetyl-lysine antibody (#9441, 1:1000/IB), LaminB1 (#13435, 1:1000/IB), and Tublin (#2148, 1:1000/IB), all from Cell Signalling Technology (Danvers, MA, USA). Mouse β-Catenin antibody was purchased from BD Biosciences (#610153; 1:1000/IB, 3 µg/IP).

The peroxidase-coupled secondary antibodies were purchased from Santa Cruz Biotechnology. Target proteins on PVDF membrane were visualised using ECL kit (Pierce, USA) and images were obtained using BioRad Imaging System (BioRad, Hercules, CA, USA).

**ChIP-qPCR assay**

The primers used for ChIP-qPCR (HDAC7 promotor) are listed below:

| Primer sequences (5′→3′) | |
| --- | --- |
|  | |
| *Region 1* | 5′-GAAAGTGGTATGCCACTTTC-3′ |
|  | 5′-TGTCCTCACGCTTCCA-3′ |
| *Region 2* | 5-GCAGCTAGCAGCCAGTCT-3′ |
|  | 5′-TGGGACTTCTTACTTCAG-3′ |
| *Region 3* | 5′-CGCTGAATATGCCGGTAGCAAC-3′ |
|  | 5′-ACCATTGTAAACCAAGGAGC-3′ |
| *Region 4* | 5′-CTCACGCTGCAGAGAGCCCAGTG-3′ |
|  | 5′-TCAGCTCAGGGCCGGC-3′ |
| *Region 5* | 5′-AGCTGAGGGAGGGCTGGAGGC-3′ |
|  | 5′-GGAAGGGTCCAGCCTGTTCCT-3′ |
| *Region 6* | 5′-AGGAACAGGCTGGACCCTTCC-3′ |
|  | 5′-CGTACACGAGCACGCATCCATG-3′ |
| *Region 7* | 5′- ATGCGAGGGTGCGTGCGCCA-3′ |
|  | 5′- GACACTGCACCGCGCGGCGCAC-3′ |
| *Region 8* | 5′- TGGCCCGCTCTCCTCAGACTCAGAT-3′ |
|  | 5′- CCAGGGGCCGGGGCCCTCAGAGC-3′ |

**Dual-luciferase assay**

β-Catenin/TCF4 transcriptional activity was measured using Super 8xTOPflash plasmid obtained from Addgene (plasmid #12456). HDAC7 transcriptional activity was measured using a luciferase assay based on the pGL3b_HDAC7 promotor-luciferase plasmid constructed from GenePharma company (Shanghai, China). pGL3-Basic, and pGL3-Basic-HDAC7 promotor wild type and mutants were constructed by GenePharma company (Shanghai, China). Cells were transfected to express the indicated proteins and with Renilla luciferase as a control for signal normalisation. Dual luciferase assays were performed according to the manufacturer’s protocol (Promega). Three independent transfections were carried out for each experiment. Data were normalised to the those for the empty vector control and are presented as average ± S.D.

**RT-qPCR**

The primers used for RT-qPCR are listed below:

| Primer sequences (5′→3′) | |
| --- | --- |
|  | |
| *ZNF326* | 5′-CAAGAGCGCATGTTGAAGGAA-3′ |
|  | 5′-CGCTGCTGCTTAATCTGGTAGA-3′ |
| *HDAC7* | 5-GGCGGCCCTAGAAAGAACAG-3′ |
|  | 5′-CTTGGGCTTATAGCGCAGCTT-3′ |
| *AXIN2* | 5′-TACACTCCTTATTGGGCGATCA-3′ |
|  | 5′-TTGGCTACTCGTAAAGTTTTGGT-3′ |
| *C-MYC* | 5′-GGCTCCTGGCAAAAGGTCA-3′ |
|  | 5′-CTGCGTAGTTGTGCTGATGT-3′ |
| *MMP7* | 5′-TCGGAGGAGATGCTCACTTCGA-3′ |
|  | 5′-GGATCAGAGGAATGTCCCATACC-3′ |
| *CCND1* | 5′-GCTGCGAAGTGGAAACCATC-3′ |
|  | 5′-CCTCCTTCTGCACACATTTGAA-3′ |
| *GAPDH* | 5′- GGAGCGAGATCCCTCCAAAAT-3′ |
|  | 5′- GGCTGTTGTCATACTTCTCATGG-3′ |

**Matrigel invasion and MTT assays**

Cell invasion assay was performed using a 24-well Transwell chamber with a pore size of 8 μm (Costar), and the inserts were coated with 20 μL Matrigel (1:3 dilution, BD Bioscience, USA). Forty-eight hours after transfection, U87 or U251 cells stably transfected with ZNF326 plasmid or ZNF326 shRNA were trypsinised and transferred to the upper Matrigel chamber in 100 μL of serum-free medium containing 5 × 10^4^ cells, and incubated for 16 h. Medium supplemented with 10% FBS was added to the lower chamber as the chemoattractant. The numbers of invaded cells were counted in 10 randomly selected high-power fields under a microscope.

Forty-eight hours after transfection, cells were plated in 96-well plates in medium containing 10% FBS at about 3 000 cells/well, and cell viability was determined by MTT (3-[4,5-dimethylthiazol-2-yl]-2, 5-diphenyltetrazolium bromide) assay. Briefly, 20 μL of 5 mg/mL MTT solution (Sigma) was added to each well and incubated for 4 h at 37°C. Then the medium was removed from each well and the resultant MTT formazan was solubilised in 150 μL of DMSO. The results were quantitated spectrophotometrically.
