# Supplementary material for: ZNF326 promotes malignant phenotype of glioma by up-regulating HDAC7 expression and activating Wnt pathway
Source: J Exp Clin Cancer Res. 2019 Jan 28;38:40. doi: 10.1186/s13046-019-1031-4 (PMC6350303; doi:10.1186/s13046-019-1031-4)
Supplement: Supplementary file 2 — Figure S1. The positive correlation between ZNF326 and HDAC7 expressions in gliomas with different grade. (A, D, G, J): Representative images of glioma specimens using H&E staining were shown (Grade I-IV, Magnification 400×). IHC staining of ZNF326 and HDAC7 expressions in gliomas with different grades outlined below: (B, C): ZNF326 and HDAC7 were both negative expressed in pilocytic astrocytoma (grade I, Magnification 400×). (E, F): ZNF326 (<25%, +) and HDAC7 (<25% positive cells) were detected in diffuse astrocytoma (grade II, Magnification 400×). (H, I): ZNF326 was nuclear positive (nearly 50%, ++), while HDAC7 (>50% positive cells, ++) was perinuclear cytoplasm positive in anaplastic astrocytoma (grade III, Magnification 400×), (K, L): ZNF326 and HDAC7 were strongly expressed (nucleus and cytoplasm, respectively, >75% positive cells, +++) in glioblastoma with grade IV (Magnification 400×). Figure S2. ZNF326 expression is associated with expression of Wnt target genes. A: Bioinformatics KEGG test was used to analyse the correlation between ZNF326 and the Wnt pathway. B: Positive correlation between ZNF326 and the four common Wnt signalling pathway target genes in glioma, analysed at the GEPIA website. Figure S3. Positive correlation between ZNF326 and HDAC7 in glioma, analysed at the GEPIA website. Figure S4. Positive correlation between HDAC7 and Wnt signalling pathway target genes in glioma, analysed at the GEPIA website. Figure S5. ZNF326 and siRNA-HDAC7 were co-transfected, or TSA (10nM) was added in U87 cells, and Transwell assays were performed to detect the changes in the invasiveness of the glioma cells. Figure S6. (A-D): ZNF326, siRNA-ZNF326, HDAC7 and siRNA-HDAC7 were transfected in U87 cells, respectively, and immunoblotting assay was performed to detect the changes in the expression of β-catenin and CK1α. GAPDH was used as a loading control. (ZIP 12193 kb) [file 13046_2019_1031_MOESM2_ESM.zip › Revised_Supplementary_Figure_legends.docx]

**Supplementary Figure S1.** The positive correlation between ZNF326 and HDAC7 expressions in gliomas with different grade.

(A, D, G, J): Representative images of glioma specimens using H&E staining were shown (Grade I-Ⅳ, Magnification 400×).

IHC staining of ZNF326 and HDAC7 expressions in gliomas with different grades outlined below:

(B, C): ZNF326 and HDAC7 were both negative expressed in pilocytic astrocytoma (grade I, Magnification 400×).

(E, F): ZNF326 (<25%, +) and HDAC7 (<25% positive cells) were detected in diffuse astrocytoma (grade II, Magnification 400×).

(H, I): ZNF326 was nuclear positive (nearly 50%, ++), while HDAC7 (>50% positive cells, ++) was perinuclear cytoplasm positive in anaplastic astrocytoma (grade III, Magnification 400×),

(K, L): ZNF326 and HDAC7 were strongly expressed (nucleus and cytoplasm, respectively, >75% positive cells, +++) in glioblastoma with grade Ⅳ (Magnification 400×).

**Supplementary Figure S2.** ZNF326 expression is associated with expression of Wnt target genes.

A: Bioinformatics KEGG test was used to analyse the correlation between ZNF326 and the Wnt pathway.

B: Positive correlation between ZNF326 and the four common Wnt signalling pathway target genes in glioma, analysed at the GEPIA website.

**Supplementary Figure S3.** Positive correlation between ZNF326 and HDAC7 in glioma, analysed at the GEPIA website.

**Supplementary Figure S4.** Positive correlation between HDAC7 and Wnt signalling pathway target genes in glioma, analysed at the GEPIA website.

**Supplementary Figure S5.** ZNF326 and siRNA-HDAC7 were co-transfected, or TSA (10nM) was added in U87 cells, and Transwell assays were performed to detect the changes in the invasiveness of the glioma cells.

**Supplementary Figure S6.** (A-D): ZNF326, siRNA-ZNF326, HDAC7 and siRNA-HDAC7 were transfected in U87 cells, respectively, and immunoblotting assay was performed to detect the changes in the expression of β-catenin and CK1α. GAPDH was used as a loading control.
