# Supplementary material for: ZNF326 promotes malignant phenotype of glioma by up-regulating HDAC7 expression and activating Wnt pathway
Source: J Exp Clin Cancer Res. 2019 Jan 28;38:40. doi: 10.1186/s13046-019-1031-4 (PMC6350303; doi:10.1186/s13046-019-1031-4)
Supplement: Supplementary file 3 — Table S1. The correlation between the expression of ZNF326 and HDAC7 in glioma. (DOCX 14 kb) [file 13046_2019_1031_MOESM3_ESM.docx]

**Supplementary table S1:**

**The correlation between the expression of ZNF326 and HDAC7 in glioma**

|  |  | ZNF326 | |  | Pearson | *P*-value |
| --- | --- | --- | --- | --- | --- | --- |
|  |  | Negative | Positive | Total | Correlation | (two-tail) |
| HDAC7 | Negative | 11 | 4 | 15 |  |  |
|  | Positive | 0 | 20 | 20 | 0.692 | 0.000* |
|  | Total | 11 | 24 | 35 |  |  |

*: statistically significant
